# Extracellular polymeric substances as local nucleation microenvironments in ureolytic MICP for concrete crack sealing: a mini review

**DOI:** 10.3389/fmicb.2026.1921241

**Published:** 2026-09-03

**Authors:** Zijun Yan, Hong Liu, Jiaqi Sun, Han Wang, Fangyu Song, Wang xiuying, Tong Chen, Xiaochen Li, Liangjing Xia

**Affiliations:** 1 Department of Pharmacy, Panzhihua Central Hospital, Sichuan, Panzhihua, China; 2 School of Pharmaceutical Sciences and Yunnan Key Laboratory of Pharmacology for Natural Products, Kunming Medical University, Yunnan, Kunming, China; 3 Department of Pharmacy, North Sichuan Medical College, Nanchong, China; 4 Faculty of Management and Economics, Kunming University of Science and Technology, Yunnan, Kunming, China; 5 School of Pharmacy, Dali University, Yunnan, Dali, China; 6 Undergraduate Division, Ocean University of China, Qingdao, China; 7 Department of Minimally Invasive Oncology, The Third Affiliated Hospital of Shandong First Medical University, Jinan, China.

**Keywords:** biofilm, calcium carbonate, concrete crack sealing, extracellular polymeric substances, microbiologically induced calcite precipitation, *Sporosarcina pasteurii*, ureolysis

## Abstract

Microbiologically induced calcite precipitation (MICP) is widely investigated for soil improvement, surface consolidation, and self-healing concrete, yet concrete crack sealing often fails not because calcium carbonate cannot form, but because precipitates are produced at poorly connected locations. In ureolytic systems, *Sporosarcina pasteurii* and related bacteria raise pH and carbonate alkalinity by hydrolyzing urea, while calcium supplies drive supersaturation. The extracellular polymeric substance (EPS) matrix around cells and biofilms is therefore not a passive coating; it can bind Ca2+, retain water and enzymes, shape diffusion gradients, and provide nucleation sites for calcite or metastable CaCO3. This mini review differs from broader MICP reviews by examining EPS control of mineral localization and bonding in concrete cracks. Representative studies report closure of 0.46-mm cracks after 100 d vs. 0.18 mm in controls and urea-hydrolysis constants of 0.09–0.91 d−1 between 10 °C and 20 °C. Evidence supports EPS effects on crystal morphology, distribution, and attachment, but causal proof within concrete cracks remains limited. We summarize how EPS chemistry and biofilm architecture affect local carbonate nucleation, how concrete crack conditions modify this process, and which measurements can separate EPS templating from bulk solution precipitation. We also compare ureolysis with other carbonate-forming pathways; adhesion, hydration, and cation binding may generalize, whereas kinetics and by-products are pathway-specific. The main implication is that MICP protocols should be evaluated by mineral localization and bonding quality, not only by total CaCO3 yield.

## 1 Introduction

Microbiologically induced calcite precipitation has become a recognizable example of how microbial metabolism can be used as a construction-relevant process. In the common ureolytic route, urease-positive bacteria hydrolyze urea, generate ammonium and carbonate species, increase local alkalinity, and promote CaCO3 precipitation when Ca2+ is available (Stocks-Fischer et al., 1999; Hammes et al., 2003; Krajewska, 2018). The approach has been explored for sand cementation, permeability reduction, limestone consolidation, heavy metal immobilization, and crack repair in concrete (Whiffin et al., 2007; Rodriguez-Navarro et al., 2003; De Muynck et al., 2010). However, concrete crack sealing is not simply a smaller version of soil biocementation. The relevant space is a narrow, chemically alkaline, intermittently wet crack, and successful healing depends on where the mineral forms as much as on how much mineral is produced.

This mini review therefore focuses on one small question: how extracellular polymeric substances around ureolytic cells influence the local nucleation microenvironment during MICP-based concrete crack sealing. EPS is usually described as a biofilm matrix composed of polysaccharides, proteins, extracellular DNA, lipids, and hydrated ions, but in MICP it also functions as a mineralization interface (Salama et al., 2016; Flemming et al., 2007; Hoffmann et al., 2021a). Earlier carbonate studies showed that bacterial outer polymers and amino acids can alter CaCO3 morphology and phase selection, while EPS-rich cells can generate crystals that differ from abiotic precipitates (Braissant et al., 2003; Tourney and Ngwenya, 2009). These findings are relevant to concrete, where the cell-EPS-mineral interface must operate under strong pH, calcium, drying, and flow constraints. In this review, “EPS-mediated nucleation” is reserved for evidence that spatially links EPS, local chemistry, and mineral initiation; mere co-occurrence of cells and CaCO3 is treated as suggestive rather than causal.

A narrow EPS perspective is useful because MICP literature often emphasizes urease activity, nutrient dosage, or final strength, whereas crack sealing also requires mineral retention at the crack wall, continuity across the crack width, and compatibility with cementitious surfaces. Unlike broad reviews that catalog bacterial strains, carriers, and macroscopic self-healing performance (De Muynck et al., 2010; Hoffmann et al., 2021a), this mini review organizes the literature around the EPS-controlled reaction microzone and the measurements required to distinguish wall-bound nucleation from bulk precipitation. Bacteria-based concrete studies have demonstrated biologically assisted crack filling, reduced permeability, and improved liquid tightness under certain conditions (Wiktor and Jonkers, 2011; Tziviloglou et al., 2016; Van Tittelboom et al., 2010; Bang et al., 2010). Yet these outcomes remain variable. This variability should not be interpreted only as a materials problem or only as a microbiological problem. Rather, it reflects a local interface in which microbial EPS, ureolytic chemistry, calcium transport, and concrete microstructure are coupled. This mini review summarizes that interface and proposes practical points for future experimental design. The scope is deliberately ureolytic; transfer to non-ureolytic pathways is evaluated explicitly in Section 2.3.

## 2 Ureolytic MICP as a localized carbonate-generating system

### 2.1 Urease-driven chemistry and the immediate cell environment

Ureolytic MICP begins with enzyme activity, but the consequences of ureolysis are local. Urea hydrolysis increases pH and dissolved inorganic carbon, and carbonate ions then react with Ca2+ when supersaturation is achieved (Omoregie et al., 2021; Zhu and Dittrich, 2016; Anbu et al., 2016). *Sporosarcina pasteurii* remains a dominant model organism because of its strong urease activity and alkaline tolerance, although engineered *Bacillus subtilis* and other urease-positive strains have also been examined (Carter et al., 2023; Yan et al., 2026; Hoffmann et al., 2021b). The reaction is often written as a simple solution process, but whole-cell studies show that cell density, enzyme localization, ammonium accumulation, and pH feedback alter precipitation rates and spatial patterns. Bulk pH, Ca2+, and ammonium profiles establish reaction progress, but they do not by themselves demonstrate an EPS effect.

The immediate cell environment is therefore an active reaction zone. Cell walls and EPS can accumulate cations through charged functional groups, while urease activity nearby supplies carbonate alkalinity. This creates a microenvironment that may reach supersaturation earlier than the bulk solution. The same mechanism can become counterproductive when rapid precipitation occurs before cells enter cracks or before reagents penetrate the target zone (Erdmann and Strieth, 2023; Wang et al., 2022). In crack repair, premature bulk precipitation wastes Ca2+, increases clogging near the surface, and leaves deeper crack segments unsealed. The practical aim is not maximum instantaneous precipitation, but precipitation that is sufficiently slow, localized, and attached to the crack wall.

### 2.2 Why crack sealing depends on mineral localization

The distinction between total CaCO3 yield and useful mineral location is central to microbial crack sealing. In soil columns, treatment efficiency can be reduced when precipitation clogs the inlet or creates non-uniform cementation, and similar spatial problems occur in cracks where the crack mouth may heal while the internal path remains permeable (van Paassen et al., 2010; Jiang and Soga, 2017; Ebigbo et al., 2012). Microfluidic studies, although usually designed for porous media, are helpful because they show bacterial attachment, detachment, aggregation, and crystal formation at pore throats or open pore bodies under flow (Wang et al., 2019; Shu et al., 2022). Concrete cracks have different chemistry, but they share the need to control the position of cells and precipitates.

EPS can help with this localization by promoting attachment and retaining water, but it can also trap ions and modify diffusion in ways that are not always beneficial. As shown in Table 1, EPS-mediated nucleation is controlled by several variables that are usually measured separately. If these variables are not distinguished, a protocol may appear successful because it produces abundant CaCO3 in solution while still failing to create a continuous mineral bridge in the crack.

**Table 1:** EPS-associated determinants of carbonate nucleation and attachment in concrete cracks.

Factor	Why it matters at the EPS-mineral interface	Possible implication for crack sealing
EPS functional groups	Carboxyl, hydroxyl, phosphate, and amino groups can bind Ca2+ and create local ion enrichment. Ca2+ binding is not uniformly promotive: at high polymer/Ca ratios, sequestration of free Ca2+ can delay nucleation.	May promote nucleation at cell surfaces or crack walls instead of only in bulk solution.
EPS amount and density	A hydrated matrix can retain enzymes, ions, and cells, but excessive density can restrict mass transfer.	Intermediate EPS production may be more useful than maximal EPS production.
Urease activity near EPS	Local carbonate alkalinity is generated where active cells or enzymes are retained.	Nucleation is favored when urease activity and Ca2+ binding occur in the same microzone. Excessive local rates can instead shift precipitation upstream of the target zone.
Cell attachment	Attached cells are more likely to seed mineral at cementitious surfaces than planktonic cells.	Improves the chance of bonded deposits and continuous mineral bridges.
Calcium source	CaCl2, calcium lactate, and cement-derived Ca2+ differ in diffusion, pH response, and compatibility. CaCl2 offers high solubility, whereas chloride-free salts trade rapid kinetics for reinforcement compatibility and possible organic-anion effects.	Controls precipitation rate, chloride risk, and interaction with EPS. For reinforced concrete, external chloride or nitrate loading and steel condition should be reported.
Moisture regime	EPS retains water but repeated drying can interrupt ureolysis and mineral growth.	Determines whether deposits become continuous or discontinuous.
Concrete surface chemistry	Portlandite, C-S-H, carbonation products, and roughness affect adhesion and nucleation.	Influences whether EPS-mineral aggregates remain attached during flow or drying.

Localization is also time dependent. Early after wetting, the important event may be cell activation and EPS hydration; during the next stage, ureolysis and calcium transport decide where supersaturation first occurs; later, crystal ripening and phase transformation determine whether deposits remain stable. Studies on biologically induced carbonate mineralization and MICP optimization indicate that precipitation kinetics, reagent concentration, and retention time strongly influence crystal size and distribution (Wang et al., 2022; Al Qabany et al., 2012; Al Imran et al., 2018). For crack sealing, this means that sampling only after visible closure may miss the microbial steps that determined the final mineral architecture.

### 2.3 Boundary of inference: comparison with non-ureolytic pathways

Ureolysis is unusually rapid because urease simultaneously generates ammonium, dissolved inorganic carbon, and alkalinity, so CaCO3 may form before cells and EPS reach the target surface. Carbonic-anhydrase routes accelerate CO2/HCO3– interconversion but still require an alkalinity or Ca2+ source; denitrification raises alkalinity under anoxic conditions but depends on electron donors and can produce N2 or N2O; sulfate reduction raises alkalinity and commonly couples strongly to EPS and sulfide chemistry; photosynthetic precipitation removes CO2 and raises pH only under illumination (Zhu and Dittrich, 2016; Braissant et al., 2007; Dupraz et al., 2004).

EPS functions such as surface adhesion, hydration, ion binding, and heterogeneous nucleation are therefore plausibly shared across pathways. By contrast, the rate-limiting step, redox requirement, gas or by-product burden, and timing of supersaturation are pathway-specific. Accordingly, the kinetic and process-design conclusions in this review apply primarily to ureolytic MICP; extrapolation to other pathways should be limited to interfacial EPS functions unless validated under pathway-specific conditions.

## 3 EPS as the nucleation microenvironment

### 3.1 Functional groups, calcium binding, and phase selection

**Figure 1:**
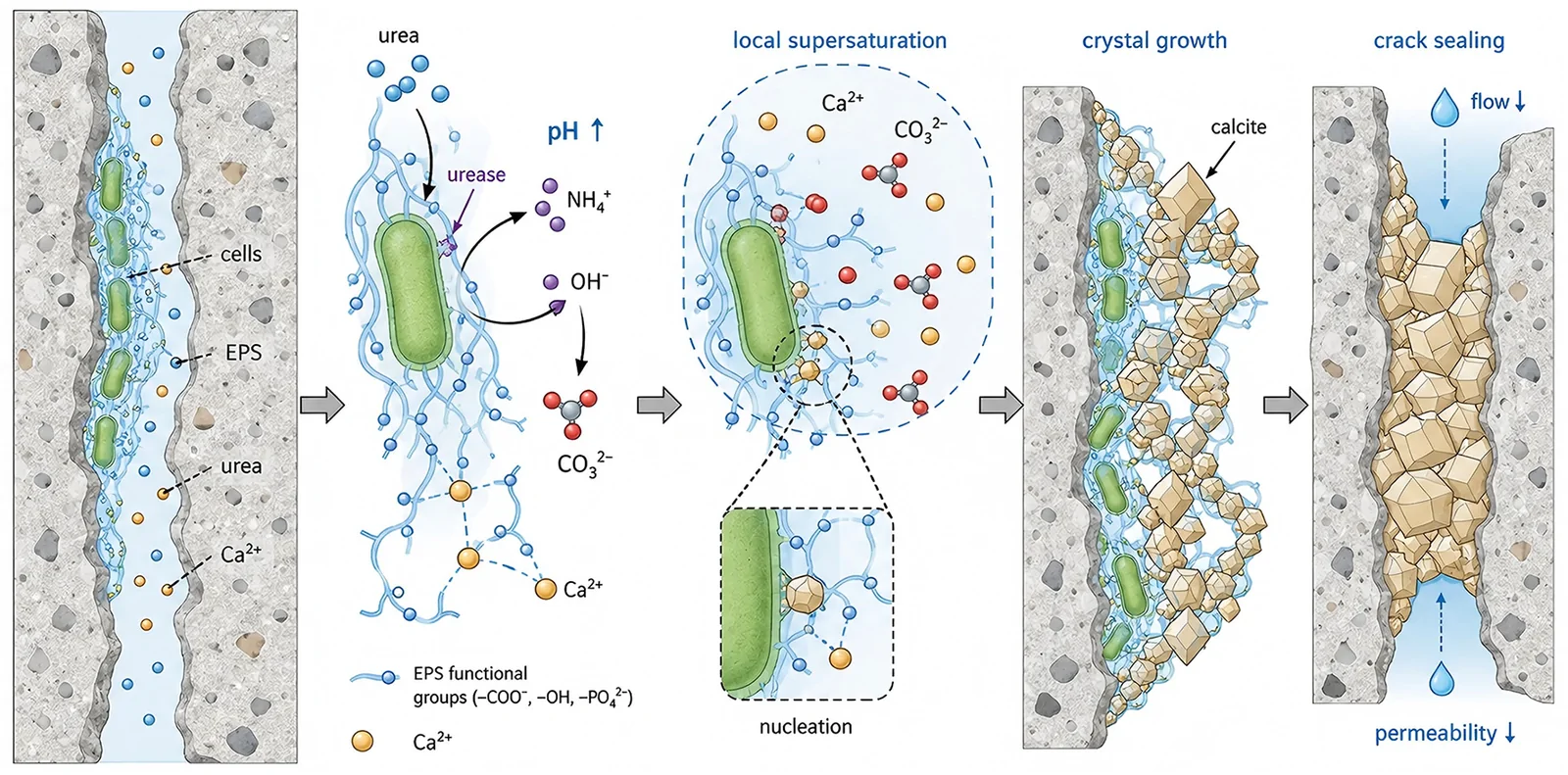
EPS-centered sequence of carbonate nucleation and crack sealing in ureolytic MICP.

EPS is important in MICP because many extracellular polymers contain carboxyl, hydroxyl, phosphate, and amino groups that can coordinate Ca2+ and alter the energetic barrier for nucleation. Studies using isolated bacterial polymers showed that extracellular materials can induce or modify calcite formation, and experiments with EPS-producing bacteria demonstrated changes in CaCO3 morphology and polymorphism (Yin et al., 2020; Szcześ et al., 2018; Hao et al., 2024). Ca2+ binding does not automatically equal promotion. At sub-saturating site occupancy, deprotonated carboxyl and phosphate groups may enrich calcium and lower the interfacial free-energy penalty; at high EPS/Ca ratios, sequestration of free Ca2+ or steric stabilization can delay nucleation. The net effect depends on charge density, polymer conformation, ionic strength, the Ca2+/EPS ratio, carbonate supply, and the distance between urease activity and bound calcium (Braissant et al., 2003; Tourney and Ngwenya, 2009; Yin et al., 2020; Szcześ et al., 2018; Hao et al., 2024). In sulfate-reducing and other carbonate-forming systems, Ca2+ bound to EPS may be released or reorganized during EPS degradation, promoting localized carbonate precipitation when carbonate species are present (Braissant et al., 2007; Dupraz et al., 2004). Direct support comes from isolated-EPS and EPS-producing-culture experiments that alter induction time, crystal organization, or polymorph. In concrete, SEM co-localization of cells, polymer-like material, and carbonate is associative; causal attribution requires EPS perturbation at matched urease activity together with time-resolved local chemistry. This does not mean that EPS always promotes the most stable mineral. Vaterite, aragonite, amorphous calcium carbonate, and calcite may appear under different biological and chemical conditions, and the stable end product depends on supersaturation, Mg2+, organic molecules, pH, and time (Meldrum, 2003; Khanjani et al., 2021; Butler et al., 2006). For concrete repair, calcite is often preferred because it is more stable and compatible with cementitious minerals, but transient vaterite or amorphous phases can still matter if they transform into better bonded calcite within the crack. A useful sequence is Ca2+ adsorption, co-localized carbonate generation, crossing of a local supersaturation threshold, heterogeneous nucleation, and maturation of an EPS-mineral composite. As shown in Figure 1, EPS should be viewed as a local organizer of ions, enzymes, and nuclei rather than as a single nucleation site.

### 3.2 Biofilm architecture, hydration, and supersaturation gradients

Biofilm architecture adds another layer of control. EPS matrices are highly hydrated and can retain enzymes, ions, metabolites, and cells within a physically heterogeneous structure (Flemming, 2016; Flemming and Wingender, 2010). In natural and engineered biofilms, pH, oxygen, nutrient, and ion gradients are common, and such gradients may allow localized mineral formation even when bulk solution measurements suggest only moderate supersaturation (Vroom et al., 1999; Stewart and Franklin, 2008). For ureolytic MICP, urease activity within or near a biofilm may produce pH microdomains, while Ca2+ sorption in EPS can create local calcium reservoirs. The mineralization pattern is then shaped by both reaction kinetics and transport through the hydrated matrix. Direct pH-gradient measurements are well-established in model biofilms (Vroom et al., 1999; Stewart and Franklin, 2008), but comparable measurements within cement cracks are scarce; a crack-scale EPS supersaturation gradient is therefore a strongly supported hypothesis rather than a routinely demonstrated fact.

These competing effects make EPS amount a non-monotonic design variable. A dense EPS matrix may support cell retention and protect cells from drying, but it may also slow reagent transport or create mineral shells that block further exchange. The most useful EPS state is likely not the highest EPS amount, but a balanced matrix that holds cells at the crack wall, maintains urease access to urea, and allows calcium and carbonate to meet at the desired location.

### 3.3 Precipitation kinetics and quantitative determinants

Precipitation rate has been measured, but cross-study comparison remains difficult because authors normalize by solution volume, biomass, urease activity, calcium conversion, or specimen mass. In artificial groundwater, first-order urea-hydrolysis constants increased from 0.09 d−1 at 10 °C to 0.91 d−1 at 20 °C, whereas peak calcite precipitation approached 0.8 mmol L−1 d−1 near critical saturation (Ferris et al., 2004). Thus, ureolysis can be strongly temperature dependent even when precipitation remains governed by saturation state.

As summarized in Table 2, the quantitative literature is non-monotonic: the highest urease activity or reactant concentration is not necessarily the condition that gives the most uniform mineral distribution. None of the cited concrete or soil studies simultaneously quantified EPS production, local saturation, precipitation rate, and connected crack fill. Table 2 therefore exposes a central evidence gap rather than implying that soil-derived optima can be transferred directly to concrete cracks.

**Table 2:** Representative quantitative and semi-quantitative evidence linking process variables to precipitation, localization, and durability.

Factor and condition	Quantitative response	EPS/localization interpretation	References
Temperature: 10, 15, and 20 °C in artificial groundwater	Urea-hydrolysis *k* = 0.09, 0.18, and 0.91 d−1; peak calcite precipitation ≈0.8 mmol L−1 d−1 near critical saturation.	Reaction rate changes sharply with temperature; EPS was not quantified, and rapid carbonate generation may outpace wall attachment.	Ferris et al., 2004
Strain activity vs. environmental adaptation	Bacillus aryabhattai urease activity was 36.55 mM min−1 (6.69% below *S. pasteurii*), yet lower-section CaCO3 precipitation was 36.22% higher.	Uniformity can improve despite slightly lower urease activity, suggesting that adaptation and retention matter; an EPS contribution was not isolated.	Wang et al., 2025
Cementation concentration, ratio, and injection cycles	Across 0.25–1.0 M, 10%−90%, and 3–21 cycles, an optimum at 0.75 M, 50%, and 21 cycles produced 14.98% CaCO3, UCS 1,030 kPa, and E50 389 MPa.	The non-monotonic optimum is consistent with transport and clogging constraints; EPS quantity/composition was not reported.	Abbasi et al., 2025; Abbasi, 2025
Freeze–thaw durability under optimized treatment	After 12 cycles, approximately 70% of UCS and 90% of stiffness were retained in MICP-treated sand.	Supports cyclic durability testing, but does not establish EPS causality or direct transfer to concrete cracks.	Abbasi et al., 2025; Abbasi and Hosseinpour, 2025
Concrete crack width and healing time	After 100 d of water exposure, bacterial concrete healed cracks up to 0.46 mm vs. 0.18 mm in controls.	Demonstrates biological engineering significance; EPS amount and depth-resolved mineral connectivity were not quantified.	Wiktor and Jonkers, 2011
Moisture regime: immersion vs. wet–dry cycling	Wet–dry treatment improved recovery of liquid tightness in bacteria-containing mortar relative to controls; no universal rate was reported.	EPS rehydration and gas/nutrient access are plausible contributors, but causal separation is still lacking.	Tziviloglou et al., 2016

k, first-order rate constant; UCS, unconfined compressive strength; E50, secant modulus. Values are study-specific and are not proposed as universal design targets.

## 4 Coupling EPS chemistry with concrete crack conditions

### 4.1 Alkalinity, ionic strength, and cementitious surfaces

Concrete cracks are selective microbial habitats rather than neutral reaction vessels. Fresh cementitious surfaces release Ca2+, OH–, and alkali ions, creating high pH and ionic strength that may support carbonate supersaturation but reduce survival of many microorganisms. Alkaliphilic or spore-forming bacteria are therefore commonly used in self-healing concrete, and nutrient carriers have been proposed to protect cells during mixing and dormancy (Jonkers and Schlangen, 2008; Wang et al., 2014; Jonkers et al., 2010). EPS may provide some local buffering and adhesion, but it cannot fully compensate for excessive alkalinity, desiccation, or nutrient limitation. The interaction between EPS and cement phases is thus a boundary condition for nucleation, not a guarantee of mineral repair. Because direct in-crack pH measurements are rare, EPS buffering in cementitious environments should be described as a proposed local effect unless measured explicitly.

Surface chemistry also affects mineral attachment. Calcium-silicate-hydrate, portlandite, unhydrated cement grains, and carbonation products provide different charge, roughness, and dissolution environments. Bacterial carbonate precipitation on degraded limestone and cementitious materials has shown that substrate properties influence mineral coating and durability (De Muynck et al., 2008a; Marvasi et al., 2020; De Muynck et al., 2008b). For crack sealing, EPS-bound nuclei must either anchor directly to these surfaces or become incorporated into growing CaCO3 bridges. If crystals grow mainly in the liquid phase and detach during drying or flow, the measured carbonate mass may overestimate functional sealing.

Another complication is that cementitious surfaces are chemically changing during healing. Carbonation, leaching, and continued hydration can alter pH and Ca2+ release while bacterial metabolism is occurring. EPS may temporarily buffer these changes by holding water and ions near the wall, but it may also become mineral-encrusted and lose biological activity. This temporal coupling is rarely captured by endpoint microscopy. Short interval sampling, even in simplified mortar cracks, would help determine whether EPS first supports adhesion and later becomes part of the carbonate-cement composite.

### 4.2 Flow, drying, and nutrient delivery at crack scale

Crack-scale transport determines whether EPS-mediated nucleation becomes a continuous seal. Flow can deliver urea, calcium, and nutrients, but it can also wash out planktonic cells and remove early precipitates. Drying can activate spore-based systems after rewetting, but it can also interrupt ureolysis and favor discontinuous deposits. These features explain why bacteria-based repair is more successful under some moisture regimes than others (Luo et al., 2022; Tziviloglou et al., 2017; Mors and Jonkers, 2019). The relevant microbial state may differ between pre-embedded healing agents, surface-applied cultures, and injection after cracking. Each mode creates a different relationship between EPS production, cell retention, and mineral location. At crack scale, the governing balance is between advection or diffusion and reaction. When ureolysis and precipitation are faster than reagent penetration, the crack mouth becomes a sacrificial reaction front. A more defensible sequence is to deliver cells first, allow residence time for wall attachment and EPS hydration, and then apply lower-concentration or alternating urea/calcium pulses. Co-injection at high concentration may maximize short-term conversion but increases entrance clogging and cell encrustation. Crack width, roughness, tortuosity, orientation, flow velocity, and dosing interval should therefore be reported as transport variables rather than merely specimen descriptors (Wang et al., 2022; van Paassen et al., 2010; Wang et al., 2019; Shu et al., 2022; Al Qabany et al., 2012). Wet–dry cycling produces competing effects: rewetting activates cells and rehydrates EPS; partial drying concentrates ions and can promote nucleation; prolonged drying arrests ureolysis and can detach or fracture poorly bonded deposits. The relevant endpoint is depth-resolved continuity after cycles, not surface closure immediately after treatment. Recent freeze–thaw studies in MICP-treated sands report substantial but incomplete mechanical retention after 12 cycles (Abbasi et al., 2025; Abbasi and Hosseinpour, 2025); these results justify durability testing but should not be extrapolated directly to reinforced-concrete cracks.

Nutrient choice also matters. Urea and calcium chloride are effective in many laboratory MICP systems, but chloride may be undesirable in reinforced concrete. Calcium lactate and organic calcium salts are frequently used in self-healing concrete, partly because they can serve as both calcium source and metabolic substrate (Jonkers and Schlangen, 2008; Jonkers et al., 2010). Calcium-source selection therefore requires a durability–kinetics trade-off. CaCl2 is inexpensive, highly soluble, and supports rapid precipitation, but exogenous chloride is unsuitable as a default for reinforced concrete because chloride accumulation can depassivate steel (Angst et al., 2009). Chloride-free calcium lactate or acetate can supply calcium and, in some systems, metabolizable carbon; their organic anions may slow precipitation or alter phase and morphology, and cost and oxygen demand must be considered (Jonkers and Schlangen, 2008; Jonkers et al., 2010). Calcium nitrate or formate avoids chloride but adds nitrate or organic ionic loads, whereas cement-derived Ca2+ minimizes added salts but is transport-limited and excessive extraction could locally decalcify the matrix. Studies should report total external chloride or nitrate, free-Ca2+ profiles, pH, and reinforcement condition rather than naming only the nominal calcium salt. EPS composition may change with nutrient conditions, so protocols that optimize urease activity alone may miss changes in adhesion and nucleation behavior. For this reason, crack repair experiments should record microbial growth state, EPS indicators, CaCO3 phase, and permeability recovery together rather than treating them as separate endpoints.

## 5 Characterization and design implications

### 5.1 Distinguishing EPS-bound nucleation from bulk precipitation

A major limitation in this area is that EPS-mediated nucleation is often inferred indirectly. Scanning electron microscopy can show crystals attached to cells or biofilms, while energy-dispersive spectroscopy confirms calcium-rich deposits, but these observations do not always prove that EPS controlled nucleation. Raman spectroscopy, X-ray diffraction, micro-computed tomography, fluorescence staining of EPS, and confocal imaging can provide complementary information about mineral phase, spatial continuity, and biofilm distribution (Butler et al., 2006; Bai et al., 2017; Janek et al., 2024). In crack specimens, permeability tests and visual crack closure should be interpreted alongside mineral localization because surface filling may not correspond to internal sealing. A minimum causal package should combine an EPS map (lectin, protein, or extracellular-DNA staining with confocal microscopy), co-registered phase or chemical mapping (Raman, micro-XRD, FTIR, or EDS), three-dimensional mineral geometry (micro-CT or serial sections), and a hydraulic outcome. SEM/EDS alone is insufficient because it cannot reliably identify hydrated EPS or establish temporal order. Localization should also be reported quantitatively. Recommended metrics are surface crack-closure ratio (1 – *At*/*A*0), median and 95th-percentile penetration depth, mineral-filled crack-volume fraction, the fraction of precipitate belonging to a wall-spanning connected cluster, mineral–wall contact area per crack volume, and hydraulic sealing ratio (1 – *kt*/*k*0, or 1 – *Qt*/*Q*0 at fixed gradient) (Hou et al., 2022). Co-registering these metrics prevents a surface crust from being misclassified as a connected seal.

As shown in Table 3, a stronger characterization strategy separates microbial state, EPS properties, solution chemistry, mineral identity, and sealing performance. Such separation avoids a common ambiguity: a treatment can fail because the bacterium was inactive, because EPS did not support adhesion, because precipitation occurred in the bulk solution, or because crystals formed but did not bridge the crack. These failures have different solutions. Increasing urea or calcium may help one case but worsen another by accelerating precipitation away from the wall. The priority column identifies the measurements required for a defensible EPS claim rather than treating every technique as equally informative.

**Table 3:** Measurements for relating EPS-mediated mineralization to crack-sealing performance.

Measurement window	Examples	Interpretation for EPS-mediated MICP	Priority for EPS claim
Microbial activity	Urease activity, cell viability, optical density, and ammonium production	Shows whether failure reflects inactive cells or poor localization of active cells.	Control
EPS quantity and composition	Total polysaccharide/protein, EPS staining, extracellular DNA, and zeta potential	Indicates whether a matrix capable of Ca2+ binding and attachment is present.	Core
Attachment and biofilm coverage	Confocal microscopy, SEM, and surface colonization assays	Helps distinguish wall-associated mineralization from suspended-cell precipitation.	Mechanistic
Solution chemistry	pH, Ca2+, urea, conductivity, alkalinity, and ammonium	Links ureolysis and supersaturation to the timing of mineral nucleation.	Core
Mineral phase and morphology	XRD, Raman, FTIR, SEM, and crystal size and shape	Determines whether stable calcite or transient phases dominate the deposit.	Mechanistic
Spatial mineral distribution	Micro-CT, polished sections, and image analysis across crack depth	Tests whether CaCO3 forms a connected seal rather than a surface crust.	Core
Functional sealing	Water permeability, sorptivity, crack closure ratio, and pressure resistance	Provides the engineering outcome that should be related back to EPS and mineral location.	Core
Durability and by-products	Wet–dry cycling, re-cracking, and ammonium/chloride monitoring	Assesses whether the biological repair is durable and compatible with concrete service conditions.	Validation

Priority definition: core measurements are required for a defensible EPS-mediated nucleation claim; Mechanistic measurements strengthen attribution; Control measurements verify biological activity; Validation measurements establish service relevance. The minimum core set is EPS mapping, local solution chemistry, three-dimensional spatial mineral distribution, and hydraulic sealing.

### 5.2 Practical monitoring and reproducibility

Reproducibility requires attention to small experimental details. Cell age, optical density, pre-growth medium, calcium source, injection sequence, retention time, crack width, curing history, and wet–dry cycle can all influence MICP outcomes (Erdmann and Strieth, 2023; van Paassen et al., 2010; Luo et al., 2015). EPS adds another variable because its amount and chemistry can shift with stress, nutrient limitation, and surface attachment. Instead of treating EPS as an uncontrolled background property, studies should report at least one EPS-related measurement, such as polysaccharide staining, protein content, total extracellular polymer yield, or microscopy-based biofilm coverage. These measurements do not need to be elaborate, but they should be paired with mineral and sealing data. Initial crack area, width distribution, tortuosity, and hydraulic conductance should be reported before treatment so that closure and sealing can be normalized across laboratories.

**Figure 2:**
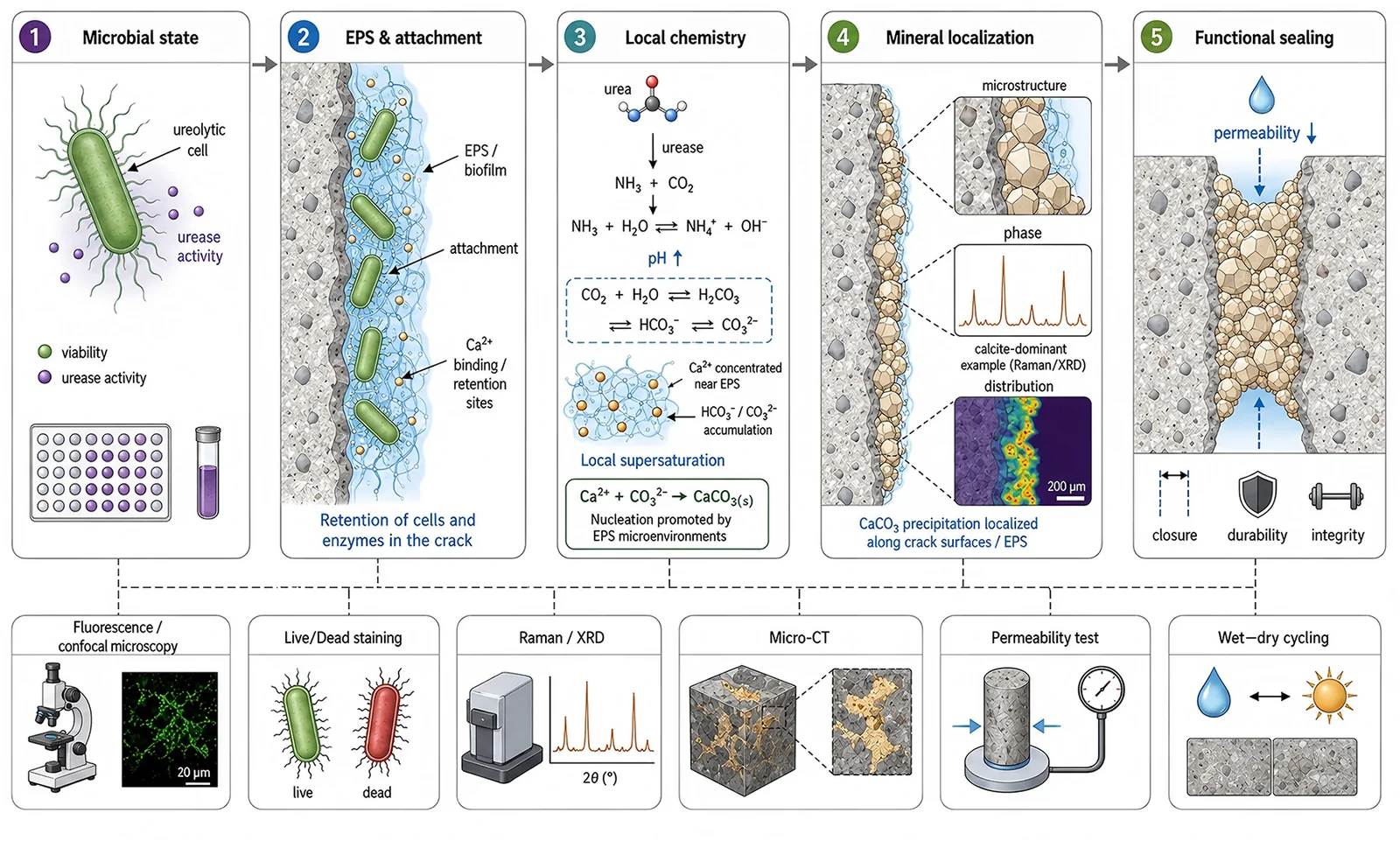
Workflow for relating EPS-mediated mineralization to crack-sealing performance.

As shown in Figure 2, a practical workflow would begin with microbial viability and urease activity, move through EPS and attachment assessment, then evaluate mineral location and crack-scale performance. This staged view helps prevent overinterpretation of single endpoints. High urease activity is useful only if cells remain in the crack; high carbonate yield is useful only if deposits are connected; and strong surface closure is useful only if permeability and durability improve. A monitoring framework of this kind can make EPS-mediated MICP more comparable across laboratories. A reference condition with reduced or removed EPS at matched urease activity is especially valuable because it converts EPS from a descriptive background variable into a testable factor.

## 6 Discussion and future directions

The available evidence supports a mechanism in which EPS modifies ureolytic MICP by concentrating calcium, shaping local supersaturation, retaining cells and enzymes, and influencing mineral morphology. This mechanism is credible across biomineralization studies, biofilm research, microfluidic MICP observations, and bacteria-based concrete repair. Evidence levels should nevertheless be separated: Ca2+ complexation and altered polymorphism are directly demonstrated in isolated-EPS or model-culture experiments, whereas EPS-driven supersaturation microdomains and improved wall bonding inside concrete cracks are usually inferred. However, the evidence is still uneven. Many studies demonstrate carbonate precipitation by ureolytic bacteria, but fewer measure EPS composition and mineral localization in the same crack-scale design. As a result, EPS is often invoked as a helpful explanation without being tested as a controllable variable.

Future studies should avoid two opposite oversimplifications. The first is to treat EPS as universally beneficial, when excessive matrix production may hinder transport or promote detached aggregates. The second is to treat MICP as purely chemical, when cell surfaces and biofilm matrices can determine where nucleation starts. More informative experiments would compare EPS-rich and EPS-reduced cell states at similar urease activity, use crack-relevant calcium sources, and monitor mineral phase transformation over wet–dry cycles. Genetically optimized bacteria may offer useful tools, but practical adoption will also require non-genetic strategies based on nutrient timing, surface conditioning, encapsulation, and moisture control (Wang et al., 2014; Zhu et al., 2024; Yu et al., 2022). EPS-mediated nucleation is therefore a small but important interface in ureolytic MICP for concrete crack sealing. It does not replace the need to control urease activity, calcium delivery, or crack moisture, but it helps explain why chemically similar protocols produce different sealing outcomes. A focused EPS-cell-mineral perspective can improve experimental interpretation and guide simpler, more reproducible repair strategies. The practical goal is not to maximize microbial mineralization everywhere, but to encourage stable carbonate formation at the crack surfaces and across the flow path where sealing is needed.

Near-term priorities are paired EPS-rich/EPS-reduced tests at matched urease activity, standardized three-dimensional localization and hydraulic metrics, chloride-free calcium-source screening, and durability protocols that include wet–dry, freeze–thaw, re-cracking, and reinforcement-corrosion exposure. Reproducibility should be assessed through interlaboratory studies using defined crack geometries and flow histories. Field implementation additionally requires shelf-life, dosing and constructability, ammonia and nutrient-discharge control, cost, worker and environmental safety, and compatibility with reinforcement. The substantial but incomplete retention after 12 freeze–thaw cycles in MICP-treated sand (Abbasi et al., 2025; Abbasi and Hosseinpour, 2025) and the available full-scale demonstrators (Mors and Jonkers, 2019) are encouraging, but they do not yet establish long-term EPS-mediated crack sealing in service.
